# Island size shapes genomic diversity in a great speciator (Aves: *Zosterops*)

**DOI:** 10.1098/rsbl.2024.0692

**Published:** 2025-03-05

**Authors:** Ethan F. Gyllenhaal, Michael J. Andersen, Robert G. Moyle, Joseph D. Manthey

**Affiliations:** ^1^ Department of Biological Sciences, Texas Tech University, Lubbock, TX, USA; ^2^ Department of Biology and Museum of Southwestern Biology, University of New Mexico, Albuquerque, NM, USA; ^3^ Department of Ecology and Evolutionary Biology and Biodiversity Institute, University of Kansas, Lawrence, KS, USA

**Keywords:** *Zosterops*, island biogeography, conservation genetics, genetic diversity, effective population size, Solomon Islands

## Abstract

Islands have long represented natural laboratories for studying many aspects of ecology and evolutionary biology, from speciation to community assembly. One aspect that has been well documented is the correlation between island size and taxonomic diversity, likely due to decreased complexity and population size on small islands. This same logic can apply to genetic diversity, which should predictably decrease with effective population size. The island size–diversity correlation has received support over the years but often focuses on single metrics of genetic diversity. Here, we use *Zosterops* white-eyes in the Solomon Islands to study the correlation between island size and various metrics related to genetic diversity, including runs of homozygosity and fixation of transposable elements. We find that almost all these metrics strongly correlate with island size, and in turn with each other. We infer that island size is independently correlated with these different variables, demonstrating that population size impacts genomic metrics of diversity in a variety of ways across temporal and hierarchical scales.

## 1. Introduction

Genetic diversity is the fundamental component of adaptation and persistence, and understanding how variation is distributed among populations is essential for understanding how natural populations evolve [1,2]. Islands serve as natural laboratories for exploring diverse topics in ecology and evolutionary biology, such as speciation and community assembly because of their discrete geographical boundaries [3–5]. Although archipelagos are characterized by dynamism, island size can be used as a proxy for population size, particularly in population genetic timescales in more stable archipelagos such as those produced by subduction zones [6]. The fact that many species effectively occupy the whole island also allows robust modelling of species ranges over time with bathymetric data and data on sea level change [7]. Thus, oceanic islands are useful as reliable proxies for estimating population size over time, which is a key factor influencing genetic diversity [8,9].

The species–area relationship [10,11] and island biogeographic theory [4,12] suggest that larger islands host greater species richness, often due to increased habitat complexity and population sizes. Island biogeographic theory also postulates that distance between regions, a proxy for connectivity, correlates with species richness. The same stochastic processes that operate on taxonomic diversity should shape genetic diversity. This relationship between island size and genetic diversity has been observed in numerous taxa and geographic contexts, such as lizards [13,14], rodents [15,16], frogs [17] and birds [18,19]. Such patterns have also been documented in continental contexts, including ‘sky islands’ [20,21] and habitat patches [22]. Many of these studies focused on a single measure of genetic diversity and few included complex but important genomic metrics (but see [23]), such as those relevant to demographic history (e.g. effective population size over time) and conservation genetic health (e.g. runs of homozygosity) [24,25].

*Zosterops* white-eyes, a species-rich bird radiation across the Afro- and Australasian tropics, present an ideal system for studying these patterns. Distributed across many Indo-Pacific islands, they have long been central to speciation theory (e.g., [3]) and discussions of the ‘great speciator’ paradox [26]. Past work has also demonstrated high population densities, even in range-restricted endemics [27]. Rapid diversification within *Zosterops* has also made relationships within this clade difficult to resolve [28–31]. The *Zosterops griseotinctus* complex is largely composed of phenotypically distinct yet genetically similar endemics in the Solomon Islands and exhibits intricate patterns of gene flow [19].

In this study, we used whole-genome resequencing of 15 species from the *Z. griseotinctus* complex to test the hypothesis that genomic diversity and other metrics of genetic variation correlate predictably with island size. We hypothesize a positive correlation between island size and (i) effective population size, (ii) genome-wide genetic diversity, and (iii) the variability of genetic diversity across the genome. Conversely, we predict a negative correlation between island size and the length and number of runs of homozygosity. Finally, we expect to see fixation of derived polymorphic transposable elements (TEs) in smaller island populations, as these generally slightly deleterious elements are more readily selected against in large populations [32,33].

## 2. Methods

### (a) Reference genome editing and transposable element annotation

We used the *Zosterops lateralis* genome [34] as a reference for this work. Because this genome was highly fragmented, we used Satsuma v. 2 [35] and the *Taeniopygia guttata* chromosome-scale genome assembly [36] to assign chromosomal coordinates to the *Z. lateralis* genome.

We annotated TEs and repetitive elements in the *Z. lateralis* genome using RepeatModeler v. 1.0.11 [37] followed by manual TE curation. RepeatModeler uses multiple programs to identify repeats: RECON [38], RepeatScout [39] and Tandem Repeats Finder [40]. We filtered sequences previously curated (≥ 98% identity) in the RepBase vertebrate database v. 24.03 [41] and created consensus sequences of novel elements with manual curation. We refined RepeatModeler consensus sequences as follows: (i) extract sequences matching de novo repetitive elements and flanking sequence with Basic Local Alignment Search Tool (BLAST) and bedtools [42,43], (ii) alignment of extracted sequences using multiple alignment using fast Fourier transform (MAFFT) [44], (iii) trim ambiguous nucleotides on edges of newly created consensus sequences, and (iv) repeat up to two times for any consensus sequences without recovered edges. We assessed any similarity of de novo elements to previously curated RepBase sequences with BLAST, using matches for naming purposes. Finally, we used the RepBase vertebrate database and newly curated repeats with RepeatMasker v. 4.08 [45] to mask and summarize repetitive and TEs in the *Z. lateralis* genome.

### (b) Resequencing and genotyping

**Figure 1.**
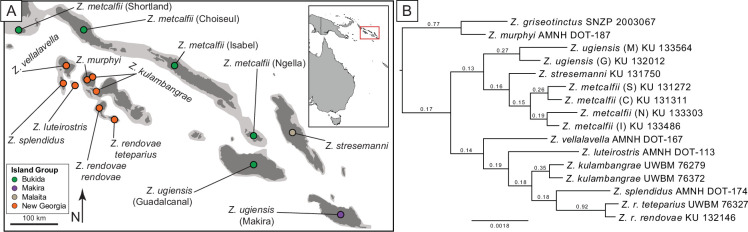
Sampling and phylogenomic relationships of *Zosterops* taxa used in this study. (A) Sampling localities for this study. Dark grey indicates current island areas and lighter grey shows approximate land connectivity at low sea levels during Pleistocene glacial maxima. (B) Maximum clade credibility tree from 40 257 phylogenies and rooted with *Zosterops simplex*. Numbers above branches show the proportion of these phylogenies supporting specific relationships, and all nodes had 100% support in the species tree with the same topology estimated in ASTRAL III.

We used a total of 17 *Zosterops* individuals in this study (electronic supplementary material, table S1), including 15 from the Solomon Islands (figure 1A), the closely related *Z. griseotinctus* and outgroup *Zosterops simplex*. The Solomon Islands samples represented nine species and 11 subspecies, with a single sample per island, excluding two sympatric species on Kolombangara (electronic supplementary material, table S1). Although the sample size per island was low, the metrics we chose were designed for single samples of similarly high depths of coverage (electronic supplementary material, figure S1), as should be representative of the population. We used DNA extractions from previous work in these taxa (e.g. [19]) to create Illumina sequencing libraries and sequenced on a NovaSeq6000 at the Oklahoma Medical Research Foundation Clinical Genomics Center. We aimed to sequence each individual at 10−20× genomic coverage, and most samples reached this expectation (electronic supplementary material, figure S1).

We used the bbmap [46] script bbduk.sh to trim sequencing adapters and quality filter raw sequencing data. We aligned reads to the reference genome using the maximal exact matches (MEM) function in Burrow-Wheeler Aligner (BWA) [47] and samtools v. 1.4.1 [48] to convert the output to BAM format and measure sequencing depth (electronic supplementary material, figure S1). We cleaned, sorted, added read groups to and removed duplicates from each BAM file using the Genome Analysis Toolkit (GATK) v. 4.1.0.0 [49]. We genotyped all individuals using three steps in GATK: (i) HaplotypeCaller function to call genotypes per individual, (ii) CombineGVCFs function to concatenate output, and (iii) GenotypeGVCFs to group genotype all individuals for variant and invariant sites. We used VCFtools v. 0.1.14 [50] to filter output genotyped sites to (i) minimum site quality of 20, (ii) minimum genotype quality of 20, (iii) minimum depth of coverage of 5, and (iv) maximum mean depth of coverage of 30. Some analyses used additional restrictions on data quality (see appropriate methods section).

### (c) Phylogenomics

We estimated phylogenies for non-overlapping 25 kbp sliding windows using RAxML v. 8.2.12 [51] with the GTRGAMMA model of sequence evolution, the most flexible model available, which allows variable substitution rates and is time reversible. Here, we filtered our data to include only biallelic and invariant sites and required a minimum of 10 000 genotyped sites to retain the window, resulting in 40 257 windows retained. From these 40 257 phylogenies, we estimated a species tree using two methods: (i) maximum clade credibility tree of all input trees using DendroPy to determine which input tree is best supported by the data [52] and (ii) the coalescent-based species tree approach ASTRAL III [53].

### (d) Genetic diversity estimates

We estimated per-individual genetic diversity as the observed heterozygosity across all genotyped sites (both variant and invariant). We estimated runs of homozygosity (ROH) per individual across 25 kbp windows with no heterozygosity and at least 80% of sites genotyped (i.e. 20+ kbp sites genotyped in 25 kbp windows).

### (e) Polymorphic transposable elements

We found 10 endogenous retroviruses (ERVs) with more than 500 copies exhibiting low divergence from TE consensus sequences (electronic supplementary material, table S2). We used these ERVs as candidates to look for between-sample TE insertion polymorphisms with the Mobile Element Locator Tool v. 2.1.2 (MELT; [54]). MELT uses split and unaligned reads from the BWA alignments, the reference genome and consensus ERV sequences to identify polymorphic ERVs. We used MELT in a multistep process: (i) discovery of potential ERVs per individual, (ii) grouping together putative polymorphic ERVs based on reference genome location, (iii) genotyping all individuals for the combined putative polymorphic ERV dataset, and (iv) filtering of all genotype calls. We ran these steps for each ERV separately, with a maximum 15% divergence from the ERV consensus sequence. Any genomic coordinates (± 100 bp) called for more than one ERV were filtered to the most similar ERV consensus sequence. We filtered the final ERV polymorphisms by removing: (i) those with imprecise breakpoints or limited evidence (MELT ASSESS flag ≥ 3), (ii) polymorphisms not passing MELT’s internal quality filters (MELT FILTER flag! = PASS), and (iii) polymorphic ERVs with greater than 25% missing data.

### (f) Demography

We estimated demographic history for each individual using MSMC2 v. 1.1.0 [55]. For use in MSMC, we masked genomic regions that were not genotyped and sites with coverage of less than eight aligned reads [56]. MSMC estimates are particularly accurate in panmictic populations, but population structure or changes in gene flow between populations may mimic changes in population sizes [57,58]. Therefore, some caution should be used when interpreting raw demographic histories. However, we largely use the demographic histories to estimate recent and harmonic mean population sizes and not strict interpretation of the population trends. In MSMC, we allowed up to 20 iterations and up to 23 distinct time segments. We performed 10 bootstrap replicates (1 Mbp genomic segments) to assess how demographic signals varied using different genomic regions. MSMC output is scaled relative to a species’ generation time and mutation rate. Here, we used double the age of sexual maturity as a generation time proxy, as in previous studies [59]. Based on an estimate of six months to sexual maturity [28], we used a 1 year generation time. We used a mutation rate of 3.16 × 10^–9^ substitutions per site per year as reported from the *Z. lateralis* genome [34].

### (g) Island size estimation

We manually measured the area of each island for all species in the Solomon Islands using satellite imagery in Google Earth. For the one montane species, *Zosterops murphyi*, we calculated the area of the species’ range by calculating the area of the highland region on the island of Kolombangara coincident with its estimated range.

### (h) Statistical analysis

We used R v. 4.4.1 [60] to conduct an array of statistical analyses. First, we visualized correlations among variables using a correlogram made with corrplot v. 0.95 [61]. Second, we performed linear regressions to examine the relationship between island size and our genetic variables. Third, due to the high collinearity of the dataset, we performed LASSO regressions with GLMNET v. 4.1-8 [62] to determine what variables were the most direct predictors of a given response variable. Although island size is an explanatory variable, to determine how it impacted different diversity metrics independently, we treated it as a response variable and all genomic variables as predictors for a LASSO regression. LASSO regressions were performed with an alpha value of 1 and lambda value selected by cross-validation.

## 3. Results

We resequenced 17 *Zosterops* individuals from the Solomon Islands at 7−16× mean coverage (electronic supplementary material, figure S1 and table S1). Using 40 257 phylogenies, we identified a species tree consistent with previous phylogenetic reconstructions using reduced-representation genomic methods (figure 1B) [19].

### (a) Demography

**Figure 2.**
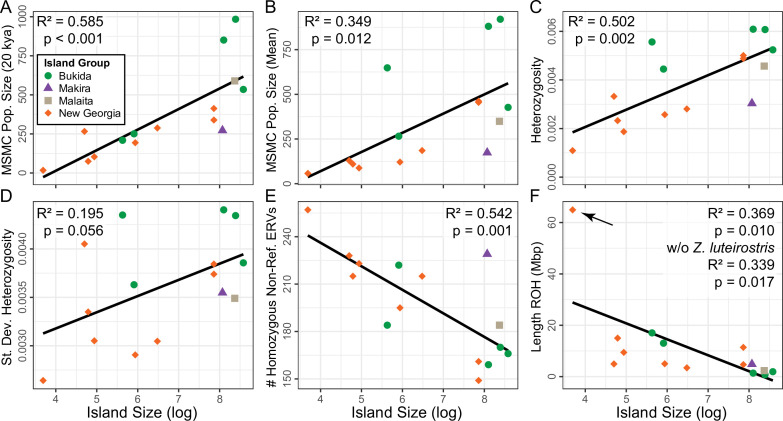
Relationships of island size and genomic parameters. Island size is strongly correlated with (A) recent and (B) harmonic mean of population size estimates in MSMC2, (C) genome-wide mean and (D) variability in heterozygosity, (E) homozygous non-reference endogenous retroviruses (ERVs) and (F) total length of runs of homozygosity (ROH). In (F), regression was performed with and without a putative outlier from the smallest island, but only the model with the outlier is shown (denoted with an arrow). Point shape and colour correspond to the island group the population is from.

Demographic history varied widely across *Zosterops* taxa in the Solomon Islands (electronic supplementary material, figure S2). Small island endemics generally have had small populations over the past 300 000 years (e.g. *N*_E_ < 200 000). Taxa with larger range sizes have experienced somewhat fluctuating population sizes through time and currently exhibit higher *N*_E_ than small island endemics (e.g. *N*_E_ ranging from 200,000 to 800,000). The demographic history of *Zosterops metcalfii* varies widely by island and likely represents both changes in population sizes and population connectivity with other island populations during Pleistocene glacial cycles. All *Z. metcalfii* populations exhibited increases in population sizes prior to 100 000 years ago. Subsequently, the population histories differed: (i) populations on the large islands of Choiseul and Isabel experienced population declines 50 000 years ago followed by population size increases; (ii) the Shortland population has drastically declined in size in the last 50 000 years; (iii) the Ngella population has generally been smaller than on the larger islands but more stable than Shortland. Recent population sizes and harmonic mean population sizes over the past 200 000 years both had a positive relationship with island size (figure 2A).

### (b) Genomic diversity

Both genomic heterozygosity and variability in heterozygosity across the genome varied widely. Individuals from smaller islands generally exhibited lower genomic diversity and less variability (although not significantly, *p* = 0.056) in diversity across the genome relative to individuals on larger islands (figure 2C,D; electronic supplementary material, figure S3 and table S1). Most metrics of genetic diversity and effective population size covaried (electronic supplementary material, figure S4). However, using a LASSO regression treating island size as a response and these genomic metrics as predictors, only non-reference ERVs, number of ROH and recent population size were included in the final model (electronic supplementary material, table S3).

The number and total length of ROH were nearly perfectly correlated (electronic supplementary material, figures S4 and S5) and higher in individuals from smaller islands (figure 2F). Notably, the *Zosterops luteirostris* individual from Ghizo, the smallest island in our sample, showed the lowest genomic diversity and a highly elevated amount of ROH (figure 2F). However, only the number and size of ROH on Ghizo were notable outliers relative to the other islands (figure 2). When considering the number and length of ROH segments, no islands strayed from a linear ratio of the two (electronic supplementary material, figure S5).

### (c) Transposable element polymorphisms

We genotyped insertion presence/absence polymorphisms for 10 recently active ERVs. We identified approximately 3000 polymorphisms with 100 to 300 homozygous ERVs absent in the reference genome per individual. Generally, individuals on smaller islands had more homozygous non-reference ERVs than individuals on larger islands (figure 2D). The number of homozygous non-reference ERVs was strongly, but not perfectly, correlated with heterozygosity (electronic supplementary material, figure S4). In a LASSO regression with non-reference ERVs as the response variable, island size, heterozygosity, historic population size and the total length of ROH segments were included as predictors in the model (electronic supplementary material, table S3).

## 4. Discussion

We demonstrated that island size is correlated with effective population size, heterozygosity and several other metrics related to genomic variability. Heterozygosity was not the strongest correlate of island size, and LASSO regression with island size did not retain it as a predictor, implying it did not explain sufficient unique variance relative to other variables (electronic supplementary material, table S3). Instead, the LASSO regression recovered that island size was most directly correlated with measures of recent population size, individual demography (i.e. ROH) and TE activity. This reflects the myriad effects that population size can have on genomes. Notably, these strong correlations arose from just modern island size, despite evidence of gene flow within and among species. However, the legacy of gene flow facilitated by glacial connectivity can still be seen in two medium-sized islands with high genetic diversity: Ngella and Shortland. Both belong to the Pleistocene Bukida group, which ranged from Guadalcanal to Shortland islands and included Bougainville (not sampled in this study). This group showed elevated diversity compared with similarly sized islands (figure 2). However, they were not outliers for recent effective population size, ROH metrics and transposon activity, which we also found as the most direct correlates of island size (figure 2, electronic supplementary material, table S3).

ROH is one of the most important statistics in the modern conservation genetics toolkit [24,25]. This statistic is a predictor of individual demography and inbreeding, and we found that island size is a predictor of ROH (figure 2F). The small island of Ghizo has extremely high ROH statistics, despite not being an outlier in any other metric (including overall genetic diversity). Additionally, its ratio between the sum of ROH lengths and the number of ROH was consistent with other populations (electronic supplementary material, figure S5). Direct inbreeding (i.e. consanguineous individuals) is expected to produce fewer longer ROH segments, while the observed pattern is consistent with a bottleneck or persistent low population size [25]. However, a bottleneck due to a founder effect is unlikely in the deeply diverged *Z. luteirostris*, and persistent low population size should manifest itself in other small New Georgia Group islands. Regardless, the high prevalence of ROH is a concern for this species’ genetic health, demonstrating the need for continued conservation attention [63]. It is worth noting that the proportion of the genome contained within ROH is low compared with other mammalian [64–66] and avian [67] systems with small population sizes. Indeed, a recent study that explored the island size–diversity relationship in smaller islands found a much higher ROH content in islands smaller than Ghizo (the smallest island in this dataset; [23]).

TEs are an important but not fully understood area of conservation genomics, as they are often weakly deleterious and may diversify in small populations, where selective forces are weaker [32]. Consistent with this hypothesis, we found that the number of homozygous non-reference ERVs was correlated with island size. This could occur due to drift (where ERVs act like neutral SNPs) and stronger selective forces against ERVs in larger populations. Drift is likely a component of this phenomenon, but does not seem to be the only one, as the negative correlation between homozygous ERVs and SNP heterozygosity is strong but imperfect (*r* = −0.83), and both island size and genetic diversity were found to be predictors in a LASSO model relating ERV fixation to other measured variables. Future work could further explore whether ERV fixation is related to other metrics of natural selection, such as the population-level synonymous and non-synonymous diversity ratio [33].

Gene flow can be a major correlate of genetic diversity [68,69], but we were not able to assess it directly in this study. However, Manthey *et al.* [19] recovered extensive gene flow among members of the Solomons *Zosterops* group, including two sympatric taxa that lack current gene flow [70]. One case of gene flow that Manthey *et al.* [19] recovered was between *Zosterops ugiensis* on Guadalcanal and *Z. metcalfi*. The impact of this gene flow can be seen in the relatively low genetic diversity of the Makira—but not Guadalacanal—population of this species. This finding is consistent with introgression from *Z. metcalfi* into *Z. ugiensis*, thereby bolstering its genetic diversity. Such gene flow may help explain why the correlation between genetic diversity and island size is not 1:1, as demonstrated by the need to use the logarithm of island size as a predictor. That relationship could be explained by Lewontin’s paradox—the empirical finding that organisms show far lower genetic diversity than would be expected based on census size [71,72]—and density compensation—where smaller islands host fewer species at higher densities [73,74].

In sum, our results emphasize the strength of island size as a predictor of a wide range of genomic variables. However, these variables are heavily correlated themselves, making it difficult to untangle true relationships. Despite this shortcoming, we still recover unique signals from different aspects of genomic diversity. Most importantly, we show that measuring multiple metrics can reveal unique aspects of evolution, as we demonstrate here in this iconic geographic radiation of white-eyes.

## Data Availability

All raw data are available on NCBI’s sequence read archive (SRA) under BioProject ID: PRJNA686795. All code to analyse the data is available at GitHub [75] and available from the Dryad Digital Repository and Zenodo at https://doi.org/10.5061/dryad.z8w9ghxqf. Electronic supplementary material is available online [76]
